# How Can Web Lessons Be Taught to Reduce Screen Fatigue, Motivational, and Concentration Problems in Different Disciplines?

**DOI:** 10.3389/fsoc.2022.871770

**Published:** 2022-05-02

**Authors:** Marina Lepp, Piret Luik, Triin Mirjam Tark

**Affiliations:** Institute of Computer Science, University of Tartu, Tartu, Estonia

**Keywords:** distance learning, students' difficulties, differences between disciplines, university students, survey, student engagement strategies

## Abstract

When conducting web lessons after transitioning to online learning due to the coronavirus, lecturers can base their work on experiences gained during the emergency situation and instructions prepared by the teaching support staff. However, students' perception of engagement strategies, screen fatigue, difficulties motivating themselves and problems with concentrating on web lessons should be also taken into account. The goal of this research is to find out how students rate the importance of engagement strategies in web lessons and how the ratings differ between disciplines. Also, the study aims to investigate how concentration difficulties, students' motivational problems and screen fatigue are connected to student engagement strategies in web lessons. To achieve that goal, 430 students of the Faculty of Sciences and Technology and the Faculty of Social Sciences at the University of Tartu were surveyed and multivariate analyses of variance and correlational analyses were conducted. Regarding student engagement strategies, “Use of slides” was found to be the most important by students. This was followed by “Explaining what and why is being studied,” “Sharing the teacher's screen,” and “Recalling what has been learned before”. The least important engagement strategies are “Presentation of article-based tasks,” “Second teacher answers questions in a chat,” and “Use of breakout rooms”. Comparing the faculties there was a statistically significant difference in the ratings given to five engagement strategies. The results showed that concentration difficulties were not related to the ratings of student engagement strategies. However, some positive correlations were found between screen fatigue and motivational difficulties, and the ratings of student engagement strategies, indicating that proper engagement strategies for conducting web lessons can be a way of influencing more students and better course design can support different needs.

## Introduction

In the spring of 2020, teaching at the University of Tartu was unexpectedly transitioned to online due to the coronavirus. As a result, teaching had to be conducted in the form of distance learning, which included web lessons. During the following semester, distance learning was partially continued, but lecturers were now able to base their work on the experiences gained during the emergency situation and instructions prepared by the teaching support staff. However, students' perceptions should be also taken into account as several studies have shown that student feedback and evaluations help teachers better plan courses and tailor them to their wishes and needs, to increase student satisfaction and quality of learning (Secret et al., 2016; Mandal, 2019). In addition, it should be considered that when staying at home, students' ability to concentrate on schoolwork and external motivation to engage in learning activities can be negatively affected by the fact that students can feel stressed, anxious, bored, and unproductive (Di Pietro et al., 2020; Scull et al., 2020). Students' attitudes toward web lessons have been studied in the past, but the results have been contradictory, which may be due to disciplinary differences. In addition, it has not been explored what student engagement strategies in web lessons are important for students who are struggling with screen fatigue and with difficulties motivating themselves and concentrating on web lessons. However, understanding the connections between fatigue, motivation, concentration, and engagement strategies is important in planning future education including web lessons and providing better learning experiences in distance learning without harming students' mental health.

## Literature Review

### Conducting Web Lessons

Conducting web lessons requires different preparation from the teacher than usual. In order to successfully organize studies, it is not enough to transfer the face-to-face learning to the web—lessons need to be adapted to the environment and possibilities, focusing on both the teaching methodology and the technology used (Guo et al., 2014; Trumm et al., 2020). Web lessons can be run both synchronously and asynchronously. In the first case, the lesson is conducted using a video conferencing application and all students are in the same (virtual) place at the same time. In the second case, the lesson is recorded in advance and students can access it through a video-sharing application. These approaches and the corresponding guidelines are very closely linked, for example, in both cases lessons should be clear, comprehensive, and engaging to avoid distractions (Mukan and Lavrysh, 2020), but there are also differences in the recommendations.

One of the main recommendations for conducting synchronous online lessons is that students should read or watch the materials at home in advance, and synchronous meetings should focus on discussing topics and consolidating what they have learned (Mukan and Lavrysh, 2020; Trumm et al., 2020; Luik et al., 2021). In order to keep the attention of students in a synchronous web lesson, they can be asked to keep the cameras on—this forces them to work more actively (Warden et al., 2013). However, as it is difficult to notice in a video when someone is preparing to ask or answer a question, limiting the number of participants can also make communication in an online lesson more convenient (Klonoski and Combs, 2009). It is recommended to use breakout rooms for group work in a web lesson, containing the optimal number of participants for the task, showing the time remaining for the work and enabling the participants themselves to enter and exit the room (Cornelius and Gordon, 2013). The need for instant messaging (chat) has also been pointed out, as it can be helpful in cases where the internet is bad or technology is failing (Bergdahl and Nouri, 2020; Mukan and Lavrysh, 2020).

Asynchronous web lessons require pre-recording of material. This is considered very important because recordings allow learners to process the material at a suitable pace (Luik et al., 2021), facilitate the acquisition of material in case of the absence (Coffey, 2010), and support independent learning (Trumm et al., 2020). There are several ways to record videos. One possibility is to record asynchronous web lessons in advance, where shorter videos are recommended at lower doses (Harrison, 2020; Massner, 2021). Synchronous web lesson can also be recorded, but in this case, their suitability must be assessed according to the content of the lesson (Cappiccie and Desrosiers, 2011) and permission must be sought to record the image and voice of the learners, as it is personal data (Trumm et al., 2020). In the case of asynchronous web lessons, there is no direct communication with students, so special attention should be paid to personality. Participants should be able to see the teacher's body language and facial expressions, and it would be good to increase eye contact (Guo et al., 2014; Gilardi et al., 2015). More enthusiastic and faster speakers help to make lessons more exciting (Guo et al., 2014). Saving a web lesson in a format where the teacher is in front of slides makes it easier for participants to focus on the slides and the teacher at the same time; in addition, a personal style, suitable animations, color and preference for images over long text blocks can all improve the presentation (Gilardi et al., 2015).

Screen sharing is considered to be a very important functionality in both synchronous and asynchronous web lessons, as it helps all parties to see the same part of the slide show simultaneously and facilitates better explanation of more complex tasks (Li, 2014). Sharing the screen with the ability to draw or write on it is also important for presentations (Correia et al., 2020). The on-screen writing and drawing function can usually be used without slides, mimicking the use of a whiteboard, which can make it easier to explain the subject (Correia et al., 2020). Freehand writing and drawing have also been shown to be more exciting than showing slides or writing code on the screen (Guo et al., 2014).

In addition to the technical side of teaching, it is also important to think about teaching methods where there is a partial overlap between web lessons and face-to-face learning. In order to take into account students' different levels and learning preferences, teachers can vary activities (Cornelius and Gordon, 2013), offer alternative tasks or provide different levels of difficulty (Mukan and Lavrysh, 2020; Luik et al., 2021) or send supportive tasks privately only to those who need them (Mukan and Lavrysh, 2020). To support learners, teachers can, at the beginning of the lesson, provide a recap of previous learning and explain what is being learned now and why (Luik et al., 2021). It is also important to engage learners, which can be done in many different ways. For example, self-assessment questions or tasks can be presented between different parts of the lesson to support learning (Harrison, 2020; Luik et al., 2021). As online lessons make it more difficult for a teacher to understand students due to a lack of non-verbal communication (Coffey, 2010), short feedback surveys on pace, material complexity, or comprehensibility could be included in suitable places (Luik et al., 2021) and learners can be often asked verbally if everything is clear (Coffey, 2010). It is also recommended to avoid giving lectures and instead allow students to contribute through presentations, comments, and interactions (Mukan and Lavrysh, 2020). The various functionalities of the applications, such as surveys, polls, hand-raising functions, emoticons, and instant messaging, can be used to increase interactivity and excitement and monitor active participation (Cappiccie and Desrosiers, 2011; Correia et al., 2020; Peper et al., 2021). It is also recommended to include regular movement or stretching breaks to increase excitement (Peper et al., 2021).

Lecturers should also take into account the specifics of the subject, as it has been found that different subjects may require the use of particular functionalities (Loch and Reushle, 2008). For example, teaching programming would benefit from the ability to share the screen and take control of the student's screen to find errors in the code more quickly (Coffey, 2010) whereas in mathematics, it is useful to mimic writing solutions on the whiteboard (Loch and Reushle, 2008).

In summary, although it is necessary to conduct web lessons differently than in face-to-face learning, advanced technology still allows students to be connected and feel involved (Li, 2014). At the same time, it must be remembered that videoconferencing alone is not a miracle—it is the lecturers who need to unlock its potential with their activities and creativity (Martin, 2005).

### Students' Perceptions of Web Lessons

The opinion of students is very important in distance learning. Decades ago, the potential of distance learning, and especially web lessons, was seen when students in a 1992 survey at the University of Iowa said that distance learning courses met their educational needs, and one student mentioned that videotape courses would be important in the future (Miller and Honeyman, 1993). Students' perceptions of distance and face-to-face learning have been repeatedly compared and different results have been obtained. In some cases, distance learning courses are preferred, where students have perceived that they are more complex and time-consuming, but at the same time teach more and are of higher quality (Hannay and Newvine, 2006). In other cases, students have considered face-to-face and distance learning courses to be equally effective (Horspool and Lange, 2012). Face-to-face meetings are also preferred because they are more interactive and engaging than distance learning and allow for a lively discussion (Klonoski and Combs, 2009).

In addition to problems with communication, problems with motivation and fatigue have been identified as shortcomings of distance learning and web lessons. Students find that distance learning makes it difficult to interact with fellow students (Horspool and Lange, 2012; Fidalgo et al., 2020) and limited interaction and socializing among students may heighten the risk of feeling isolated (Ferri et al., 2020; Putri et al., 2020). In addition, when learning at home students can feel stressed and unproductive, and this can negatively affect their ability to concentrate on learning activities, stay present while taking web classes, and lower motivation (Di Pietro et al., 2020; Peper et al., 2021). In an Active Minds survey among college students, 85% of students reported that focusing on school has been the most difficult thing and 80% of respondents said that COVID-19 has negatively impacted their mental health (Active Minds, 2020). Furthermore, students can experience congestion (Sowan and Jenkins, 2013) and loss of attention, especially when they are multitasking (Peper et al., 2021). Distance learning and web lessons also increase the time spent in front of screens (Dushkevych et al., 2020), which is tiring and exhausting for students (Trumm et al., 2020; Oducado et al., 2021). During synchronous web lessons, students also noticed the new phenomenon of feeling exhausted during or after video conferencing, known as videoconference fatigue or “Zoom fatigue” (Bailenson, 2021).

According to students, teachers can do a lot to improve web lessons. Students feel that engagement and perceived learning are most influenced by the presence of the teacher, including humor and personal examples (Hibbert, 2014). Student engagement strategies like allocating time for student questions is considered beneficial in synchronous communication with a teacher (Abou-Khalil et al., 2021), and questions asked during web lessons are considered to support learning (Harrison, 2020). In addition, it has been suggested that asynchronous web lessons can improve students' overall experience and satisfaction with distance learning where synchronous learning does not take place, as they allow closer contact with the teacher and are more similar to face-to-face learning (Harrison, 2020). This also increases engagement and motivation (Scagnoli et al., 2019).

### Aim and Research Questions

It appears that students' perceptions of distance learning and web lessons have been studied in the past, but the results have been mixed. This may be due in part to differences in disciplines. The goal of this research is to find out how students from different disciplines rate the importance of engagement strategies in web lessons in relation to students' concentration difficulties, motivation problems, and screen fatigue. Three research questions were posed:

1. What student engagement strategies are rated as more important by students, and what aspects receive lower importance ratings?
2. How do the ratings of engagement strategies differ when comparing students from two different disciplines?
3. How are concentration difficulties, motivational problems, and screen fatigue of students from different disciplines related to their ratings of engagement strategies?

## Materials and Methods

### Study Context

The University of Tartu, where the study was carried out, had quite a long track record in distance learning even before the coronavirus pandemic situation. In 1991, the first video lecture was recorded at the university, and since 1998 online learning environments for conducting lectures and workshops have been used (TÜ e-õpe ajajoonel). After that, distance learning has been applied in online in-service training (42.9% of fully web-based online in-service training courses in 2019) and also in regular education, with 69% of fully or partially web-based courses in 2019 (Pilt, 2020).

However, the coronavirus pandemic that began in 2020 affected distance learning at the University of Tartu. A case study (Trumm et al., 2020) found that the number of distance learning courses in the Moodle environment did not increase significantly due to the emergency, but the number of Panopto video lectures and BigBlueButton webinars increased significantly. According to the authors, the reason for the increase was the introduction of digital solutions in order to conduct lessons in a situation of forced distance learning. In the same study, it was found that both teachers and students generally coped with the situation.

### Sample

The sample of the study consisted of the students of the Faculty of Sciences and Technology and the Faculty of Social Sciences at the University of Tartu, representing two different disciplines [STEM (science, technology, engineering, and mathematics) and social science], who answered the questionnaire. A total of 430 students from the University of Tartu formed the sample (325 of them rated all engagement strategies and they are used in the comparison test). Of these, 199 (46.3%) studied at the Faculty of Sciences and Technology and 231 (53.7%) at the Faculty of Social Sciences including 2 colleges. Narva College is a part of the Faculty of Social Sciences, which includes divisions of Civic Studies, Psychology and Pedagogy, Estonian Language and Literature, Russian Language and Literature, and Foreign Languages. Pärnu College is a part of the Faculty of Social Sciences, which includes departments of Entrepreneurship, Tourism Studies and Social Work Administration. All the institutes with teaching responsibilities were represented in the study and the distribution of respondents by institute is presented in Table 1.

**Table 1:** Distribution of respondents by institute and faculty.

**Faculty of sciences and technology**		**Faculty of social sciences**	
**Institute**	**Respondents**	**Institute**	**Respondents**
Computer Science	73 (17.0%)	Education	63 (14.7%)
Ecology and Earth Sciences	36 (8.4%)	Narva College	52 (12.1%)
Molecular and Cell Biology	28 (6.5%)	Law	33 (7.7%)
Technology	24 (5.6%)	Pärnu College	28 (6.5%)
Mathematics and Statistics	14 (3.3%)	Psychology	24 (5.6%)
Physics	13 (3.0%)	Social Studies	15 (3.5%)
Chemistry	11 (2.6%)	Political Studies	11 (2.6%)
		Economics and Business Administration	5 (1.2%)
Total	199 (46.3%)	Total	231 (53.7%)

Two hundred and seventy-seven of the respondents (64.4%) were bachelor students, 125 (29.1%) were master's students, 23 (5.3%) were applied higher education students and 5 respondents (1.2%) were integrated bachelor's and master's students. Three hundred and twenty-one of the respondents (74.7%) were women, 100 (23.3%) were men, and nine respondents (2.1%) did not want to disclose their gender. Regarding age, 13.5% of the sample were aged up to 19, 48.8% were aged 20–24, 10.7% were aged 25–29, 6.3% were aged 30–34 and 20.7% were aged 35 and over.

### Data Collection and Analysis

**Figure 1:**
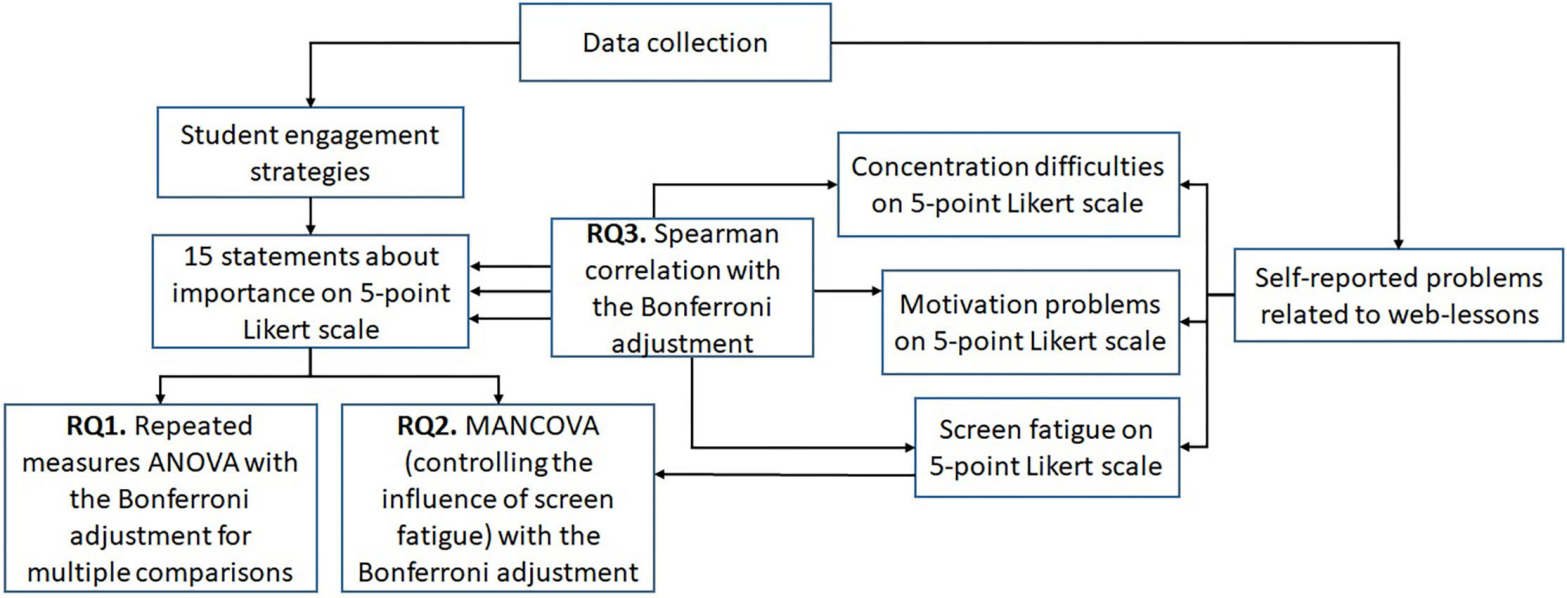
Data collection and analysis.

Data were gathered using an anonymous questionnaire, which was administered to the students of the Faculty of Sciences and Technology (FST) and the Faculty of Social Sciences (FSS) of the University of Tartu through the mailing lists of respective institutes in December 2020. Respondents had to rate 15 items on a 5-point Likert scale [ranging from a score 1 (“not important at all”) to 5 (“very important”)], indicating the student engagement strategies that they consider important when participating in web lessons (see Figure 1). The items were composed according to previous studies (Klonoski and Combs, 2009; Sowan and Jenkins, 2013; Luik et al., 2021). Also, students rated their own perceptions of using screens and web lessons with three items: (1) it is harder for me to concentrate; (2) it is harder for me to motivate myself, (3) I feel screen fatigue. The prefacing statement to all these items was “Participating in web lessons” and the items were rated by students on a 5-point Likert scale (1-totally disagree … 5-totally agree). The questionnaire ended with a background data section.

To ensure validity of the questionnaire, two students from the sample piloted it before it was distributed. The students in the pilot study were a 3rd-year undergraduate from the Institute of Computer Science and a 3rd-year undergraduate from the Institute of Psychology, thus representing the Faculty of Sciences and Technology and the Faculty of Social Sciences, respectively. The activities of the students were monitored while participating in the pilot study in filling in the questionnaire and more specific questions were asked if necessary. As the questionnaire was not significantly modified after the piloting, the answers of these two students were also included in the sample.

Statistical analyses were carried out as follows (see Figure 1): First, descriptive statistics on students' ratings of the importance of student engagement strategies and perceptions of students about difficulties were investigated. Answering the first research question, the multivariate analyses of variance (repeated measures ANOVA) with the Bonferroni adjustment for multiple comparisons were used to identify differences between the ratings of the different strategies. For the second research question, the MANCOVA multivariate analysis of variance test was used to compare the ratings of student engagement strategies between students from the two faculties, controlling the influence of screen fatigue. In the last phase, Spearman correlation coefficients were calculated to investigate the relationship between concentration difficulties, students' motivational problems, screen fatigue and the ratings of student engagement strategies for web lessons. As the aim was to know which ratings of 15 student engagement strategies are related to the evaluations on concentration difficulties, we used Bonferroni correction dividing *p*-value to 15. The same division was conducted with the evaluations on motivational problems and evaluations on screen fatigue.

## Results

### The Most and the Least Important Student Engagement Strategies

Descriptive statistics of the student engagement strategies are presented in Table 2. As the values for asymmetry and kurtosis between −2 and +2 are considered acceptable in order to prove normal univariate distribution (George and Mallery, 2010), all the strategies indicated the univariate normality of the data and a parametric test was used. There was a statistically significant difference between the ratings of different strategies [*F*_(1, 328)_ = 11,431.263, *p* < 0.001, η2 = 0.735]. Multiply comparison with Bonferroni adjustment indicated that four engagement strategies (“Use of slides,” “Explaining what and why is being studied,” “Sharing the teacher's screen,” and “Recalling what has been learned before”) were evaluated significantly higher than the other strategies (comparison from the others all *p* < 0.05). There was also a statistically significant difference between the strategies “Use of slides” and “Recalling what has been learned before” (*p* < 0.01), but there were no other statistically significant differences between these four engagement strategies (all *p* > 0.05).

**Table 2:** Descriptive statistics of ratings of student engagement strategies.

**Engagement strategies**	***M***	**SD**	**Mdn**	**Sk**	***K***
Use of slides	4.21	0.888	4	−1.266	1.842
Explaining what and why is being studied	4.04	1.031	4	−1.088	0.733
Sharing the teacher's screen	4.02	1.050	4	−0.970	0.189
Recalling what has been learned before	3.90	0.951	4	−0.799	0.379
Giving self-assessment questions or tasks to test knowledge	3.60	1.133	4	−0.607	−0.305
Conduction of short feedback surveys (about tempo, material complexity, comprehensibility)	3.47	1.140	4	−0.344	−0.654
Giving time to read slides	3.45	1.158	4	−0.389	−0.606
Taking a break from thinking	3.32	1.098	3	−0.242	−0.710
Pre-recording a lecture and answering questions during the web class	3.40	1.284	4	−0.370	−0.915
Drawing or writing on slides or shared screen	3.21	1.194	3	−0.038	−0.985
Taking stretching breaks	3.19	1.390	3	−0.119	−1.284
Offering a selection of tasks of varying difficulty	3.28	1.172	3	−0.238	−0.822
Use of breakout rooms	2.78	1.247	3	0.061	−1.021
Second teacher answers questions in a chat	2.85	1.268	3	0.084	−1.048
Presentation of article-based tasks	2.54	1.209	2	0.408	−0.745

*M, Mean; SD, Standard Deviation; Mdn, Median; SK, skewness; K, Kurtosis*.

Of all other engagement strategies, “Presentation of article-based tasks,” “Second teacher answers questions in a chat,” and “Use of breakout rooms” were evaluated significantly lower (with Bonferroni adjustment all *p* < 0.05). There was also a statistically significant difference between the strategies “Presentation of article-based tasks” and “Use of breakout rooms” (*p* < 0.01), but there were no other statistically significant differences between these three engagement strategies (all *p* > 0.05).

### Differences in the Ratings of Engagement Strategies Between Students From Two Different Disciplines

At first, we present descriptive statistics for difficulties as perceived by students in web lessons (Table 3). Again all aspects showed skewness and kurtosis values between the −2 and 2 indicating the univariate normality of data. Comparing perceptions of students about experiences of screen fatigue, low motivation, and low concentration in web lessons from the two faculties with *t*-test, there was no statistically significant difference in the case of statements about concentration [*t*_(427)_ = 0.499, *p* > 0.05, *d* = 0.048] and motivation [*t*_(427)_ = −0.306, *p* > 0.05, *d* = −0.030]. However, a statistically significant difference was found in the statement about screen fatigue [*t*_(423)_ = 5.734, *p* < 0.01, *d* = 0.558], which was higher ranked by students from the Faculty of Social Sciences (*M* = 3.77, SD = 1.284) compared with the students from the Faculty of Science and Technology (*M* = 3.04, SD = 1.333).

**Table 3:** Descriptive statistics of ratings of difficulties of using screens and web lessons.

**Difficulty**	***M***	**SD**	**Mdn**	**Sk**	***K***
Concentration difficulties	3.35	1.368	4	−0.378	−1.055
Motivation problems	3.37	1.360	4	−0.370	−1.122
Screen fatigue	3.43	1.351	4	−0.410	−1.048

*M, Mean; SD, Standard Deviation; Mdn, Median; SK, skewness; K, Kurtosis*.

As the significant difference was found in the case of screen fatigue and this aspect could influence ratings on student engagement strategies, we used MANCOVA controlling by the screen fatigue ratings. Comparing the ratings given by students from the two disciplines, a significant statistical difference appeared in the case of five student engagement strategies, with *F*_(15, 308)_ = 11.245, *p* < 0.001, Wilks' Λ = 0.646 and partial eta-squared η2 = 0.254. “Sharing the teacher's screen” and “Drawing or writing on slides” were rated higher by the students of the Faculty of Sciences and Technology, while the statements “Conduction of short feedback surveys (about tempo, material complexity, comprehensibility),” “Taking stretching breaks,” and “Use of breakout rooms” were rated higher by the students from the Faculty of Social Sciences (see Table 4).

**Table 4:** Comparison of ratings of student engagement strategies between students from the two disciplines.

**Engagement strategies**	**FST (*****n*** **= 147)**		**FSS (*****n*** **= 178)**		**Comparison between FST and FSS**			
	***M***	**SD**	***M***	**SD**	***F***	***p***	**df**	**η2**
Use of slides	4.24	0.878	4.18	0.903	1.094	0.296	1	0.003
Explaining what and why is being studied	4.03	0.965	4.04	1.093	0.069	0.793	1	<0.001
Sharing the teacher's screen	4.17	0.982	3.92	1.065	6.301	0.013^*^	1	0.019
Recalling what has been learned before	3.87	0.909	3.92	0.991	0.031	0.860	1	<0.001
Giving self-assessment questions or tasks to test knowledge	3.60	1.157	3.60	1.117	0.428	0.513	1	0.001
Conduction of short feedback surveys (about tempo, material complexity, comprehensibility)	3.24	1.144	3.65	1.106	4.596	0.033^*^	1	0.014
Giving time to read slides	3.30	1.113	3.58	1.163	1.981	0.160	1	0.006
Taking a break from thinking	3.18	1.058	3.45	1.090	2.742	0.099	1	0.008
Pre-recording a lecture and answering questions during the web class	3.36	1.287	3.43	1.279	0.147	0.702	1	<0.001
Drawing or writing on slides or shared screen	3.45	1.160	2.99	1.188	14.946	<0.001^***^	1	0.044
Taking stretching breaks	2.46	1.251	3.82	1.170	84.836	<0.001^***^	1	0.209
Offering a selection of tasks of varying difficulty	3.21	1.142	3.34	1.193	0.057	0.812	1	<0.001
Use of breakout rooms	2.19	1.119	3.30	1.118	67.745	<0.001^***^	1	0.174
Second teacher answers questions in a chat	2.87	1.299	2.85	1.251	0.525	0.469	1	0.002
Presentation of article-based tasks	2.46	1.212	2.64	1.194	0.013	0.910	1	<0.001

*M, Mean; SD, Standard Deviation; df, degrees of freedom; η2, partial eta-squared*.

* *p < 0.05,*

*** *p < 0.001 multiple comparisons with Bonferroni correction*.

### Relationship Between Screen Fatigue, Motivational and Concentration Difficulties, and Student Engagement Strategies

There were no significant relationships between concentration difficulties and the ratings of student engagement strategies, neither in the case of students from the Faculty of Sciences and Technology nor from the Faculty of Social Sciences (Table 5). However, the results indicated that the ratings of the strategy “Offering a selection of tasks of varying difficulty” was significantly related to the students' evaluations on motivation problems for students from both disciplines. The ratings of student engagement strategy “Presentation of article-based tasks” was significantly correlated with the students' evaluations on motivation problems only in the case of students from the Faculty of Sciences and Technology. The ratings of engagement strategies “Explaining what and why is being studied” and “Conduction of short feedback surveys (about tempo, material complexity, comprehensibility)” were significantly related to the evaluations on motivation problems only in the case of students from the Faculty of Social Sciences. As the third difficulty, relations of ratings of students' engagement strategies with the evaluations on perceptions on screen fatigue were explored. In the case of students from both faculties, evaluations on screen fatigue were significantly related to the ratings of the strategy “Presentation of article-based tasks”. In addition, in the case of students from the Faculty of Sciences and Technology, significant correlations with the ratings of “Taking a break from thinking” and “Taking stretching breaks” were found.

**Table 5:** Spearman correlation coefficients.

**Engagement strategies**	**Concentration difficulties**		**Motivation problems**		**Screen fatigue**	
	**FST**	**FSS**	**FST**	**FSS**	**FST**	**FSS**
Use of slides	−0.055	0.094	−0.062	0.073	0.068	0.120
Explaining what and why is being studied	−0.026	0.165	−0.046	0.199^*^	0.028	0.167
Sharing the teacher's screen	−0.127	−0.010	−0.139	0.024	0.060	0.111
Recalling what has been learned before	0.030	0.067	0.079	0.141	0.102	0.126
Giving self-assessment questions or tasks to test knowledge	0.003	0.127	0.108	0.154	0.126	0.135
Conduction of short feedback surveys (about tempo, material complexity, comprehensibility)	0.105	0.159	0.137	0.213^*^	0.149	0.171
Giving time to read slides	0.047	0.047	0.093	0.048	0.202	0.035
Taking a break from thinking	0.088	0.020	0.198	0.088	0.276^*^	0.091
Pre-recording a lecture and answering questions during the web class	0.066	0.027	0.108	0.062	−0.019	0.069
Drawing or writing on slides or shared screen	0.076	0.000	0.078	0.096	0.047	0.054
Taking stretching breaks	0.107	0.046	0.103	0.077	0.228^*^	0.122
Offering a selection of tasks of varying difficulty	0.186	0.145	0.228^*^	0.226^*^	0.080	0.106
Use of breakout rooms	−0.040	0.051	−0.041	0.049	0.058	0.059
Second teacher answers questions in a chat	0.079	0.059	0.085	0.019	0.029	0.155
Presentation of article-based tasks	0.123	0.146	0.239^*^	0.097	0.241^*^	0.228^*^

* *p < 0.05, for multiple correlations Bonferroni adjustment was used*.

## Discussion and Conclusion

The first research question sought to identify the student engagement strategies that receive, respectively, higher and lower ratings from students. The students found four engagement strategies to be significantly more important than others. The use of slides and the sharing of the screen, which are closely related, were among the important student engagement strategies. Screen sharing together with slide shows has been considered important by students in the past (Coffey, 2010; Li, 2014). Thus, it can be concluded that teachers should continue showing slides and sharing the screen in web lessons, as this is highly appreciated by students. Other engagement strategies that were considered important were explanations of what is being learned and why, and reiterations of previous learning. It is recommended to do both at the beginning of the online lesson (Luik et al., 2021), and students have previously emphasized that web lessons should facilitate consolidation of what has been learned (Trumm et al., 2020), which is also related to the mentioned student engagement strategies. Therefore, it is important for students that teachers help them understand and consolidate what they are learning.

Three student engagement strategies—“Presentation of article-based tasks,” “Second teacher answers questions in a chat,” and “Use of breakout rooms”—received significantly lower ratings than others. It is quite logical that presentation of article-based tasks was the least important engagement strategy for students as it was found earlier that merely transferring face-to-face learning (and article-based tasks as part of it) online is not enough—web lessons should use different teaching methodology and be adapted to the environment and possibilities (Guo et al., 2014; Trumm et al., 2020). Secondly, receiving answers to questions by the second teacher in a chat was not considered important by students although chat has been previously pointed out as a helpful aspect (Bergdahl and Nouri, 2020; Mukan and Lavrysh, 2020). We can assume that maybe teachers allocated separate time for questions as it was found to be beneficial (Abou-Khalil et al., 2021) and all questions were answered verbally by the teacher. In addition, students did not consider the use of breakout rooms to be particularly important for their studies. This may indicate an unfavorable attitude toward group work as previously a negative attitude toward group work has emerged in a study by Sowan and Jenkins (2013) where students felt separated from group members while working in groups during distance learning. However, this can also be caused by the specifities of the subject or the environment used. As group work is a common teaching method and the use of breakout rooms is recommended (Cornelius and Gordon, 2013), further research is needed into why students do not find the use of breakout rooms to be important in web lessons and what teachers could do to improve it.

As the second part of the first research question, we discuss how the ratings of student engagement strategies differ when comparing students from two different disciplines. The statements regarding sharing of teacher screen and drawing or writing on slides or shared screen were rated higher by STEM students compared to social sciences students. The increased desire to see the teacher's screen may be partly due to the fact that the more technical subjects taught to STEM students require seeing the topic (code, formulas etc.) and hearing precise explanations in order to grasp it. This finding is consistent with previous research, which found that more complex tasks can be better explained when screen sharing is used as it allows students and the teacher to see the same part of the slide show at the same time (Li, 2014). In addition, it has been observed that the ability to share the screen is beneficial in teaching programming, which is one subject of STEM (Coffey, 2010). The importance of the ability to write or draw on screen, mimicking the use of a whiteboard, has also been mentioned in connection with STEM, specifically mathematics (Loch and Reushle, 2008).

Social science students gave higher ratings to statement about the use of breakout rooms. The subjects taught in social sciences contain more communication, discussion, and cooperation which can be better conducted using breakout rooms, with an optimal number of participants for the task (Cornelius and Gordon, 2013), and that is why it is natural for this aspect to be more important for students in social field. Since there is quite a big difference in the ratings of the use of breakout rooms, this may have been the reason why the overall average for the use of breakout rooms was quite low. It seems that, based on students' opinions, breakout rooms are useful in some areas, but not universally. As there are several suggestions on how to increase excitement and monitor active participation in web lessons (Cappiccie and Desrosiers, 2011; Correia et al., 2020; Peper et al., 2021), it seems that strategies such as asking feedback or including stretching breaks are more important for social science students compared to STEM students. This difference is probably due to the inherent specificities of the two disciplines. In STEM areas there is also laboratory work, which includes in natural way movement and hands-on activities and therefore activation of students is already built into the learning process. However, in a social field, there is more theoretical material and thus it is necessary to use techniques that can activate learners.

Our last research question was intended to find how the difficulties to concentrate, students' motivation and screen fatigue are connected to the ratings of the student engagement strategies for web lessons. The results showed that only concentration difficulties were not related to the ratings of student engagement strategies. However, there were found two significant relationships between motivation problems and the ratings of student engagement strategies in the case of students from science and technology discipline and three significant relationships in the case of students from social sciences. As all found correlation coefficients were positive, it can be assumed that several engagement strategies are rated as more important by students who find that it is hard for them to motivate themselves in web lessons. Offering a selection of tasks of varying difficulty is important for students with motivation problems from both disciplines. Giving alternative tasks or with different levels of difficulty have been considered previously as means to support learners (Mukan and Lavrysh, 2020; Luik et al., 2021). Interestingly, only students from social sciences who reportedly have more motivation problems tended to give higher ratings to two student engagement strategies—explaining what and why is being studied and conduction of short feedback surveys (about tempo, material complexity, comprehensibility). The last one, this kind of feedback from students, inserted in suitable places, might help teachers to understand students' needs and adapt their teaching to the need of learners (Luik et al., 2021). In opposite, the ratings of presentation of article-based tasks correlated only with the ratings of students from the science and technology field who reported higher levels of motivation problems.

Several positive correlations were found in the case of screen fatigue which indicates that some engagement strategies are rated as more important by students who reportedly have more screen fatigue. It seems that the presentation of article-based tasks is important for students from both disciplines who report that they suffer on screen fatigue. However, taking stretching breaks and taking a break from thinking were more important only for students from the science and technology field who reported higher levels of screen fatigue. It might be that learning in the science and technology field is more intensive and therefore breaks are needed to avoid exhaustion from the screen.

Our findings can be useful for lecturers of different disciplines, as a better vision of the engagement strategies important to students and their correlation with students' motivation and screen fatigue might help to inform the course design to better support different needs. As the results of the study revealed several differences between the fields, these differences should be brought into sharper focus in the future and recommendations distributed to lecturers could be diversified based on the particular field. At the same time, the research provides valuable information for teachers as they prepare web lessons, enabling them to keep in mind the engagement strategies that are really important for students.

As a limitation of the research, it could be pointed out that only students from two fields at a single university were surveyed, and some institutes were under-represented. In the future, a more extensive study could be conducted among students of all fields at the University of Tartu and the ratings of students from several different universities could be compared. Further, lecturers could also be asked to give their opinion. In addition, the students' answers did not distinguish between synchronous and asynchronous web lessons, so only general results were obtained.

## Data Availability Statement

The raw data supporting the conclusions of this article will be made available by the authors, without undue reservation.

## Ethics Statement

Ethical review and approval was not required for the study on human participants in accordance with the local legislation and institutional requirements. The patients/participants provided their written informed consent to participate in this study.

## Author Contributions

ML, PL, and TT contributed to conception and design of the study and wrote sections of the manuscript. TT organized the database. PL performed the statistical analysis. ML wrote the first draft of the manuscript. All authors contributed to manuscript revision, read, and approved the submitted version.

## Conflict of Interest

The authors declare that the research was conducted in the absence of any commercial or financial relationships that could be construed as a potential conflict of interest.

## Publisher's Note

All claims expressed in this article are solely those of the authors and do not necessarily represent those of their affiliated organizations, or those of the publisher, the editors and the reviewers. Any product that may be evaluated in this article, or claim that may be made by its manufacturer, is not guaranteed or endorsed by the publisher.
